# Mysterious giants in the world of lipids: long linear isoprenoid functions in plant physiology and reproduction

**DOI:** 10.1093/jxb/eraf481

**Published:** 2025-10-31

**Authors:** Małgorzata Gutkowska, Ewa Swiezewska, Joanna Szewińska, Joanna Rojek, Liliana Surmacz

**Affiliations:** Department of Biochemistry and Microbiology, Institute of Biology, Warsaw University of Life Sciences, Warsaw, Poland; Institute of Biochemistry and Biophysics, Polish Academy of Sciences, Warsaw, Poland; Department of Biochemistry and Microbiology, Institute of Biology, Warsaw University of Life Sciences, Warsaw, Poland; Department of Plant Biology and Biotechnology, Faculty of Biology, University of Gdańsk, Gdańsk, Poland; Institute of Biochemistry and Biophysics, Polish Academy of Sciences, Warsaw, Poland; RIKEN Center for Sustainable Resource Science, Japan

**Keywords:** Dolichol, GPI anchor, isoprenoids, membrane biophysical properties, protein glycosylation, sexual plant reproduction, solanesol, ubiquinone

## Abstract

Isoprenoids, sometimes also called terpenoids, form a large and diversified family of natural compounds. All isoprenoids are synthesized from a branched five-carbon isoprene unit, which is derived in plants in the cytoplasmic/endoplasmic reticulum (ER)/peroxisome mevalonic acid (MVA) pathway and plastidial methylerythritol phosphate (MEP) pathway. The isoprene unit modules in isoprenoid molecules may be arranged in various ways, the relatively simplest being iterative head-to-tail condensation. Long-chain linear polymers derived in this manner are present in plastidial membranes, where they play a role in photosynthesis. Long-chain linear isoprenoid polymers synthesized on the MVA pathway are also found in the ER membrane (dolichols) and mitochondrial inner membrane [*all-trans*(*E*)-nonaprenol trivial name solanesol or *all-trans*(*E*)-decaprenol trivial name spadicol]. Both dolichol and solanesol play crucial roles in eukaryotic metabolism: dolichol serves as a cofactor in protein glycosylation and glycosylphosphatidylinositol (GPI) anchor synthesis, while solanesol (or spadicol) acts as the side chain of ubiquinone in electron transport during cellular respiration. Some new roles of the long-chain linear isoprenoid compounds in plants may also be anticipated based on *in vivo* work on yeast and *in vitro* biophysical experiments. This review focuses on the known and emerging roles of these compounds in the context of plant physiology and sexual reproduction.

## Teaser

Dolichol and solanesol are long-chain isoprenoid lipids with crucial functions in all eukaryotes. This review focuses on the specific roles of dolichol and solanesol in the physiology, development, and sexual reproduction of plants.

## Introduction

### Long-chain linear isoprenoids

**Fig. 1.**
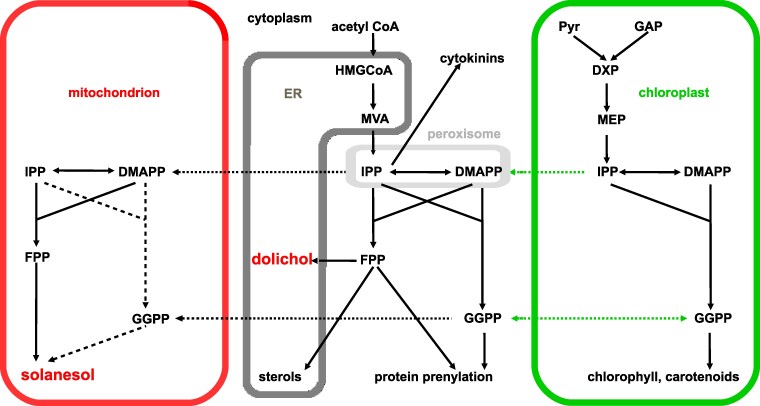
Schematic representation of isoprenoid metabolism in a plant cell. The main isoprenoid-biosynthesizing pathways in chloroplast, mitochondria, and cytoplasm/ER/peroxisomes are depicted, with the names of central metabolites. Solid arrows show the well-established metabolic routes, and dotted arrows depict the controversial steps. The green rectangle on the right side of the scheme represents a chloroplast, the red rectangle on the left side of the scheme represents a mitochondrion, the dark gray shape represents ER cisternae, and the light gray shape represents a peroxisome. Ac-CoA, acetyl-coenzyme A; HMG-CoA, 3-hydroxy,3-methyl-glutaryl-coenzyme A; MVA, mevalonic acid; Pyr, pyruvate; GAP, glyceraldehyde 3-phosphate; DXP, 1-deoxy-D-xylulose 5-phosphate; MEP, 2-C-methyl-D-erythritol 4-phosphate; IPP, isopentenyl diphosphate; DMAPP, dimethylallyl disphosphate; FPP, farnesyl diphosphate; GGPP, geranylgeranyl diphosphate.

Isoprenoids are natural compounds of incredible diversity in the plant kingdom. Most of them serve in plant secondary metabolism, and their functions include repelling predators, attracting pollinators, communicating with the rhizosphere, and eliminating unicellular pathogens (reviewed by Bergman *et al*., 2024). Despite these species-specific functions, isoprenoids take part in the primary metabolism of plants, functioning as membrane modifiers (sterols), protein lipid anchors (farnesyl and geranylgeranyl group), cofactors of glycosylation (dolichols), electron carriers in the respiratory chain and photosynthesis (ubiquinone, plastoquinone, and phylloquinone), photosynthetic pigments (carotenoids and chlorophylls), antioxidants (tocopherols), and finally hormone precursors (brassinosteroids, abscisic acid, cytokinins, and strigolactones) (Fig. 1). Most isoprenoids are relatively small molecules, consisting of 10–30 carbon atoms; however, several groups of isoprenoids consist of much longer chains, sometimes counting >20 isoprene units (corresponding to 100 carbon atoms) arranged in a linear fashion.

### Basic processes of angiosperm sexual reproduction

Since dolichol and solanesol (or spadicol) play crucial roles in eukaryotic metabolism, their proper function is required in sexual plant reproduction, with the emphasis on male gametophyte development and pollen–pistil interaction. In order to better understand the functions of dolichol and long-chain *all-trans*(*E*)-prenols and their derivatives in plant reproduction and development, it is necessary to recall some basic facts about gametophytes and seed formation in *Angiospermae* plants.

**Fig. 2.**
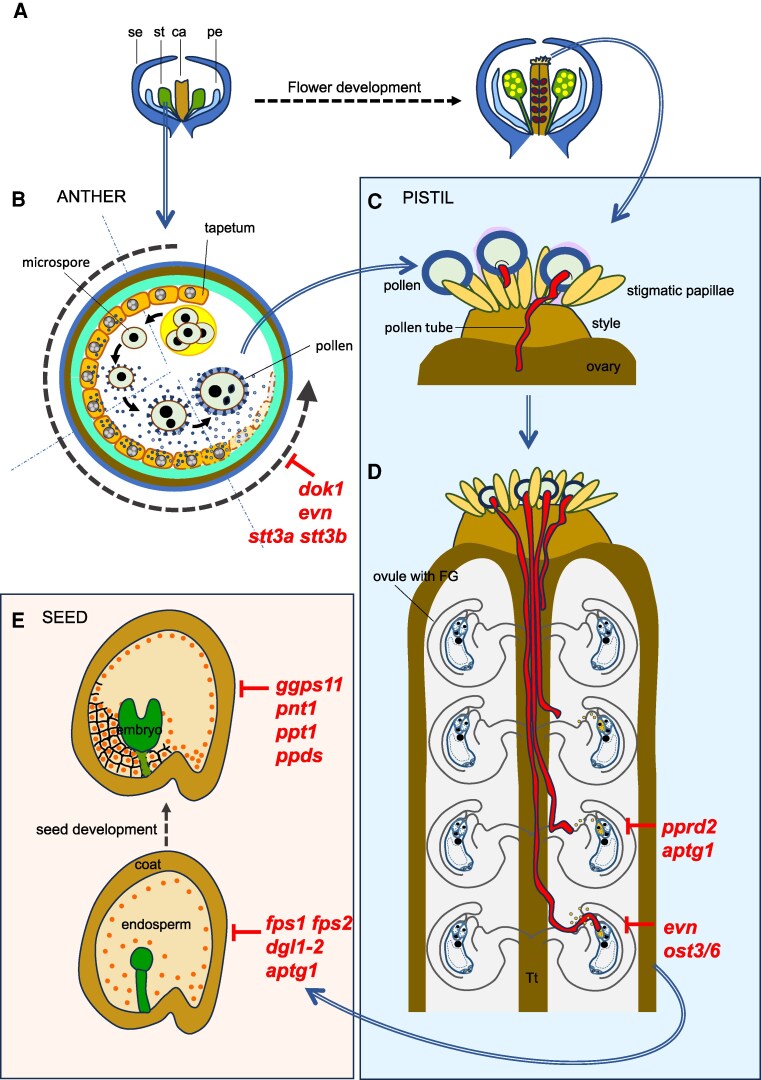
Schematic representation of the influence of the failure of isoprenoid-dependent processes on angiosperm generative development. Developmental stages at which development of knockout mutants in isoprenoid biosynthesis genes stops are marked on the scheme; a detailed description is given in Table 1. (A) Flower development; (B) male gametophyte development; the arrows show the direction of development over time from the post-meiotic tetrad of microspores through unicellular and bicellular pollen grain to the mature tricellular pollen grain that is released upon anthesis. In parallel, the tapetum layer in the anther nourishes developing pollen and finally degenerates to form the pollen coat. (C) Pollination on the stigma; pollen grains recognized by the stigma as compatible hydrate and form pollen tubes. (D) Pollen tube growth through the transmitting tract, guidance to the micropyle, pollen tube rupture, and fertilization of the egg cell and central cell of female gametophyte by two pollen tube-derived male gametes. Consecutive stages are shown from top to bottom of the scheme. (E) Seed development; the embryo differentiates from the fertilized egg cell and the endosperm differentiates from the fertilized central cell. The arrow shows the direction of changes over time. Abbreviations: se, sepals; st, stamens; ca, carpel; pe, petals.

The reproductive stage in the development of flowering plants includes the formation of the male and female gametophytes, pollination, and fertilization (Fig. 2). Though some plants avoid meiosis and fertilization (apomictic plants; Tucker and Koltunow, 2009), almost all produce gametes and finally form a functional seed. The male gametophyte, called a pollen grain, develops from precursor cells (the microsporocytes) inside the pollen sac, located deep within the anther tissue (Fig. 2A). The microsporocyte undergoes meiosis, leading to the formation of four haploid microspores arranged in tetrads. Microspores released from the tetrads continue to develop through first and second mitosis, eventually forming the three-cell-containing male gametophyte. During these processes, the specialized pollen wall is formed. The tapetum, the layer adjacent to the pollen sac, plays an essential role in the formation of pollen wall (Fig. 2B). The pollen wall protects the male gametophyte and allows communication with the stigma (Fig. 2C). The pollen wall is built by an inner intine, an outer exine, and a pollen coat layer; the latter two layers are mainly composed of lipids. Both the tapetum and the gametophyte contribute to pollen wall formation; the tapetum provides the components of the exine (mainly sporopollenin) and pollen coat, and the pollen grain contributes to the intine (Lou *et al*., 2014; Ischebeck, 2016; Hafidh and Honys, 2021). The task of the mature male gametophyte is the transport of two male gametes and their discharge into the female gametophyte (Fig. 2C, D). Inside the ovule, double fertilization takes place, and the embryo and endosperm form as a consequence of this (Fig. 2D, E). After pollen–stigma recognition, the compatible pollen hydrates, and this leads to pollen tube germination. Pollen tubes elongate within the pistil to find the ovules through several steps: stigma penetration; elongation into the transmitting tract; emergence from the transmitting tract; funicular guidance; and micropylar guidance (Fig. 2D; Mizuta and Higashiyama, 2018). The precise growth of pollen tubes towards the ovules depends on a combination of mechanical, chemotropic, geometrical, and physiological guidance signals. In Arabidopsis, the transmitting tract is lined with an extracellular matrix rich in polysaccharides, glycolipids, and abundant arabinogalactan proteins (Mizuta and Higashiyama, 2018; Pereira *et al*., 2021). After pollen tube emergence from the transmitting tract, the next step is pollen–ovule communication. This interaction involves many signals emitted from the female gametophyte that reorient pollen tube growth toward the micropyle of the unfertilized ovule and ultimately guide the pollen tube inside one of the egg-cell-accompanying cells, the synergids, to rupture and release the sperm cells (Mizuta and Higashiyama, 2018; Broz and Bedinger, 2021). Fertilization includes adhesion and fusion of male and female gametes. Both processes must be precise and require the action of molecules on the surface of the gametes (Sprunck, 2020). Double fertilization leads to the development of embryo and endosperm. At the same time, a seed coat forms from the ovule integuments. After fertilization of the egg cell by the sperm cell, the resulting zygote elongates and divides further to form the embryo proper and suspensor (Fig. 2E). The fertilization of the central cell gives rise to the endosperm. The suspensor connects the embryo proper to the surrounding nourishing maternal tissues. At the transition to the heart stage of embryo development, the embryo epidermis expresses sucrose transporters, which allow direct uptake of nutrients from the endosperm while the suspensor gradually degenerates. Coordination of the endosperm cellularization process with the transport of nutrients to the embryo is crucial for the further development of the embryo and mature seed (Lafon-Placette and Köhler, 2014).

### Two pathways for isoprenoid biosynthesis in plants—a track of ancient microorganisms in eukaryotic cells

Coming back to isoprenoid lipids, they consist of a branched hydrocarbon chain and a polar head group. In Bacteria, bactoprenols (long isoprenoid alcohols) serve as obligatory cofactors in the synthesis of oligosaccharides of cell walls and are indispensable for cell survival (Caffalette *et al*., 2020). In Archaea, isoprenoids are the major membrane-building lipid components, forming an ether bond with glycerol instead of fatty acid glycerol esters in other organisms (Jain *et al*., 2014). The isoprenoid-built archaeal membranes are more stable in extreme environments and ensure high thermostability and low ion permeability to the cells (Koga, 2012). Bacterial isoprenoids are synthesized on an MEP (methylerythritol phosphate) metabolic pathway, using pyruvate and glyceraldehyde-3-phosphate (GAP) as precursors (Xue and Ahring, 2011), while archaeal isoprenoids are derived from a (modified) MVA (mevalonic acid) metabolic pathway, using acetyl-CoA as a precursor (Lombard and Moreira, 2011). The abundance of non-cyclic isoprenoids in eukaryotic cells is much lower than in microorganisms, but they are still indispensable cell components. Plants are unique eukaryotes in that they preserve both pathways for isoprenoid biosynthesis, in contrast to animals, which rely solely on the MVA (mevalonic acid) pathway (Hemmerlin *et al*., 2012; Pu *et al*., 2021) (Fig. 1). In mature plants at standard growth conditions, the exchange of isoprenoid precursors between plastids and cytoplasm is limited; however, in very young seedlings and under stress conditions, some flow of the MEP-derived precursors to the cytoplasm has been noted (Rodriguez-Concepcion *et al*, 2004; Opitz *et al*., 2014; Jozwiak *et al*., 2017; Lipko *et al*., 2023). (Fig. 1). The plastidial pool of metabolites cannot substitute for the absence of the cytoplasmic isoprenoid synthesis in the processes of development of gametophytes and seeds; knockout mutations in MVA pathway genes cause plant male sterility or embryolethality (Okada *et al*., 2008; Suzuki *et al*., 2009; Ishiguro *et al*., 2010; Kobayashi *et al*., 2018).

## Dolichols

Dolichols are long-chain isoprenoid alcohols present in all eukaryotic cells. They are of different lengths with a branched hydrocarbon backbone, ranging from several up to 30 isoprenoid units, which make them one of the largest hydrophobic molecules in the cell (Sagami *et al*., 2018). In comparison with sterols, their much more abundant biosynthetic relatives, the properties and functions of dolichol in the membrane are still obscure. The amount of dolichols in the cell is approximately several μg g^–1^ DW, 100–1000 times lower than that of sterols, which are present in amounts >1 mg g^–1^ DW (Kaliszewski *et al*., 2008; Segatto *et al*., 2014; Jozwiak *et al*., 2017; Lipko *et al*., 2023). Dolichols, which are extremely hydrophobic, are obligatorily membrane bound, but relatively little is known about the structural changes their large molecules force on the lipid bilayer. What is more, even in humans, the biosynthetic route leading to dolichols has only recently been fully elucidated (Wilson *et al*., 2024).

Dolichyl phosphate is the obligatory factor in the *O-* and *N-*linked glycosylation of eukaryotic proteins (in plants, not in *O-*glycosylation, which proceeds through a strikingly different mechanism, without engagement of dolichol-phosphate sugar donors; Schoberer *et al*., 2018) as well as glycosylphosphatidylinositol (GPI) biosynthesis (De Coninck *et al*., 2021). The presence of *N*-glycans on glycoproteins influences protein activity, stability, and function. *N*-glycosylation is a key player in the quality control of protein maturation and trafficking (Saijo *et al*., 2009; Haweker *et al*., 2010). GPI-anchored proteins are crucial factors in cell–cell recognition, in particular in a multicellular context of specialized tissues and organs. Important physiological processes based on glycoprotein recognition are host–pathogen or host–symbiont interactions, plasma membrane–cell wall–extracellular matrix anchoring, and many others (a recent summary on glycoprotein functions in eukaryotes and the evolution of glycosylation processes is presented in Varki, 2011, 2017, and in plant–pathogen recognition in Soni *et al*., 2024; other processes Strasser, 2018, 2022; Strasser *et al*., 2021; plant development is comprehensively reviewed in De Coninck *et al*., 2021). In particular, glycan–glycan interaction is crucial in pollination and gamete recognition during fertilization (Capron *et al*., 2008; Feng *et al*., 2019; Li *et al*., 2023 ; Figueiredo *et al*., 2024). Less studied functions of dolichols may include shaping membrane biophysical properties and, specifically in plants and fungi, impregnation of vulnerable spores (or possibly other organs, e.g. roots) against harsh environmental conditions.

### Dolichol synthesis

**Fig. 3.**
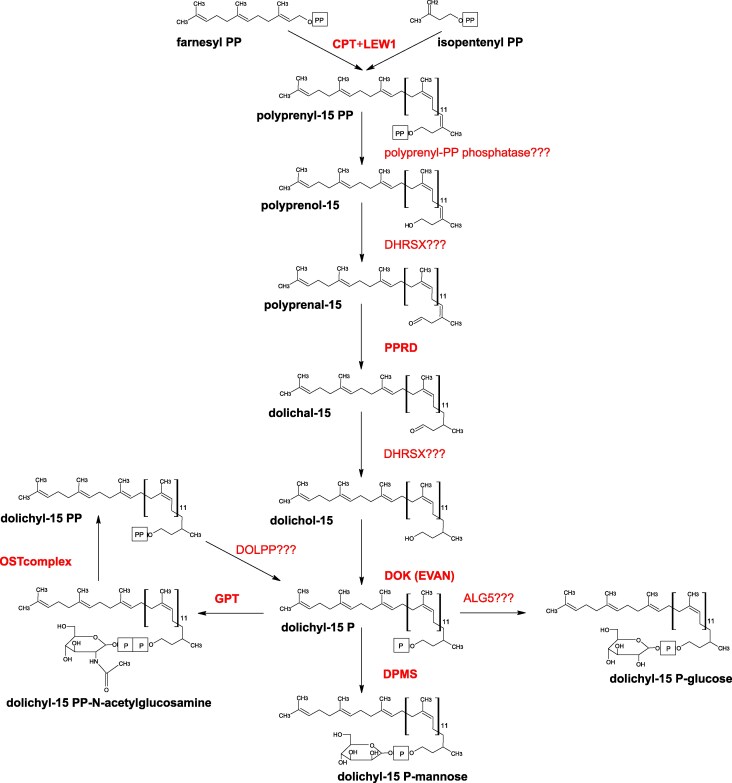
Biochemical pathways involved in dolichyl phosphate glycoside and dolichyl diphosphate oligosaccharide synthesis and dolichol recycling. For clarity, only isoprenoids and related metabolites are shown with full chemical formulae. Full names of the enzymes and EC numbers are summarized in Table 1. Picture prepared in ChemSketch.

Dolichyl phosphate is synthesized in a few consecutive reactions: condensation of isoprenoid blocks of isopentenyl diphosphate (IPP) starting from a farnesyl diphosphate (FPP) to form a polyprenyl diphosphate, which is dephosphorylated, and reduced (in a three-step reaction) on a double bond closest to the hydroxyl group to form dolichol. Subsequently, a pool of dolichol becomes phosphorylated to form dolichyl phosphate (Fig. 3) (Wilson *et al*., 2024; summarized in Grabińska *et al*., 2016), which can be modified further by sugar moieties.

#### Isopentenyl diphosphate synthesis

In the first reaction on the pathway leading to dolichol, a short *all-trans*(*E*)-allylic precursor, FPP, is formed. It is synthesized from five-carbon isoprenoid molecules, IPP and its isomer dimethylallyl diphosphate (DMAPP) (Figs 1, 3). These molecules come from the MVA pathway or the MEP pathway, as described in the preceding paragraphs. The pool of IPP and DMAPP stays in equilibrium due to the action of the enzymatic activity of IPP delta isomerase enzyme (IDI). In plants, this enzyme is localized to peroxisomes, mitochondria, and plastids, providing distinct, but to some extent interchangeable, pools of isoprenoid precursors (Okada *et al*., 2008; Phillips *et al*., 2008; Sapir-Mir *et al*., 2008; Jin *et al*., 2020). In Arabidopsis, one IPP isomerase (IDI1L) is targeted to plastids, the other (IDI2L) is targeted to mitochondria, and IPP isomerase activity encoded by both genes (IDI1S and IDI2S) is present in the peroxisomes (Phillips *et al*., 2008; Sapir-Mir *et al*., 2008). All data concerning gene names and loci, enzyme classification, and the influence of mutation on the development of Arabidopsis are summarized in Table 1. Single knockout mutants in *IDI* genes in Arabidopsis are viable and fertile; however, the *idi2* mutant flower is deformed with sepals fused and petals underdeveloped (Phillips *et al*., 2008). The double mutant is lethal; however, the detailed analysis of the reason behind the lethality has not been described. From genetic segregation analysis, it might be assumed that both male sterility and embryo lethality are responsible for the null genetic transmission (Okada *et al*., 2008; Phillips *et al*., 2008) (Fig. 2). The synthesis of IPP/DMAPP in each compartment and transport of IPP between the three compartments must be regulated to satisfy the metabolic needs of particular downstream compounds in the cell. Moreover, the translational control of the protein localization of IDI enzymes depends on the cell/tissue demand in changing conditions (Phillips *et al*., 2008; Sapir-Mir *et al*., 2008).

**Table 1.** Summary of enzymatic functions and phenotypic manifestations of mutants in isoprenoid metabolism genes in Arabidopsis

Enzyme; EC no.	Locus in Arabidopsis	Isoform	Male gametophyte	Female gametophyte	Seed	Reference
IDI; EC 5.3.3.2, isopentenyl diphosphate isomerase	At5g16440	IDI2L-chloroplast	Single mutants have fertile pollen	Normal genetic transmission of single mutation; no male transmission of double mutation	Single mutants viable, no double mutants	Phillips *et al*. (2008); Okada *et al.* (2008); Sapir-Mir *et al.* (2008)
		IDI2S-peroxisome				
	At3g02780	IDI1L-mitochondria				
		IDI1S-peroxisome				
FPS; EC 2.5.1.10, farnesyl diphosphate synthase	At5g47770	FPS1L-mitochondria	Single mutants have fertile pollen; ∼2% genetic transmission of *fps1 fps2* double mutation; *fps1 fps2* pollen grains mature and germinate, pollen tube grows shorter than the wild-type	Normal genetic transmission of single and double mutation	Normal in single mutants; >50% embryo abortion of *fps1 fps2*, embryo halted at octant/early globular stage	Closa *et al.* (2010)
		FPS1S-cytosol				
	At4g17190	FPS2-cytosol				
GGPS; EC 2.5.1.29, geranylgeranyl diphosphate synthase	At1g49530	GGPS1-mitochondria	Normal genetic transmission			Ruiz-Sola *et al.* (2016a)
	At2g18640	GGPS3-cytosol	No data			
	At2g23801	GGPS4-cytosol	High expression in anthers; no genetic or developmental data			
	At4g36810	GGPS11S-cytosol	Normal genetic transmission	Normal genetic transmission	25% of embryos halted at transition from globular to heart stage	Ruiz-Sola *et al.* (2016b)
CPT1; EC 2.5.1.87, *cis-*prenyltransferase	At2g23410	CPT1-ER	No data		No homozygous progeny	Surowiecki *et al.* (2019)
CPT3; EC 2.5.1.87, *cis-*prenyltransferase	At2g17570	CPT3-ER	RNAi lines fertile pollen	RNAi lines fertile FG	RNAi lines homozygous seeds viable	Gawarecka *et al.* (2022)
CPT4; EC 2.5.1.87, *cis-*prenyltransferase	At5g60510	CPT4-ER, lipid bodies	High expression in anthers	No data	Homozygous seeds viable	Surmacz and Swiezewska (2011); Surowiecki (2022)
CPT5;EC 2.5.1.87, *cis-*prenyltransferase	At5g60500					
LEW1; *cis-*prenyltransferase accessory protein (common subunit for CPT3, CPT4, and CPT5)	At1g11755	LEW1-ER	No data		No homozygous progeny; embryo lethality	Brasher *et al.* (2015); Zhang *et al*. (2008); Surowiecki *et al.* (2019)
PPRD1; EC 1.3.1.94, polyprenal reductase	At1g72590	PPRD1-ER	No data		Normal	Jozwiak *et al.* (2015); Wu *et al*. (2025)
PPRD2; EC 1.3.1.94, polyprenal reductase	At2g16530	PPRD2-ER	0% genetic transmission through male gametophyte, pollen viable, pollen tubes grow *in vitro* but often burst	No data	No homozygous progeny	Jozwiak *et al.* (2015)
DOK1; EC 2.7.1.108, dolichol kinase	At3g45040	DOK (dolichol kinase)	0% genetic transmission through male gametophyte; pollen grains degenerate at tricellular stage; RNAi lines fertile pollen	28% genetic transmission; pollen tube often does not burst in ovary; RNAi lines fertile FG	No homozygous progeny	Kanehara *et al.* (2015); Lindner *et al.* (2015)
DPMS1; EC 2.4.1.83, dolichol-phosphomannose synthase	At1g20575	Part of DPMS complex	Viable	Viable	Seeds wrinkled, cell wall defects	Jadid *et al.* (2011)
DPMS2	At1g74340		No data			
DPMS3	At1g48140					
GPT; EC 2.7.8.15, GlcNAc-1-phosphotransferase	At2g41490	GPT also known as GDPAT1; ALG7 homolog, ER	No data		No data, seedlings overexpressing GPT resistant to tunicamycin	Koizumi *et al.* (1999)
PPDS; EC 2.5.1.85, *trans-*polyprenyl diphosphate synthase	At2g34630	SPS (COQ1)-dual plastid/mitochondria localization	No data		16% embryolethality	Ducluzeau *et al.* (2012); van Schie *et al.* (2007)
PPT1; EC 2.5.1.39, *trans-*polyprenyl transferase	At4g23660	HBPDT (COQ2; HRL1)-mitochondria	No data		Embryo lethality at the transition from globular to the heart stage of development	Okada *et al.* (2004)

Only genes expressed in generative organs are included. Only enzymes with non-plastidial localization were included.

#### Farnesyl diphosphate synthesis

IPP and DMAPP molecules condense to form a *trans*(*E*)-prenyl diphosphate. If one molecule of DMAPP reacts with two molecules of IPP in consecutive reactions catalyzed without dissociation of the intermediate, a 15-carbon-long di-*trans*(*E*)-farnesyl diphosphate is formed (Fig. 3). FPP synthases (FPSs) are enzymes present in the cytoplasm and mitochondria (Cunillera *et al*., 1997). The cytoplasmic isoform contributes to the synthesis of sterols, brassinosteroids, dolichols, and farnesyl groups for protein prenylation, while the mitochondrial isoform produces solanesol, the side chain of ubiquinone. No chloroplast isoform has been described so far.

In Arabidopsis, FPS enzymes form a small family, with FPS1S and FPS2 proteins targeted to the cytoplasm, while the FPS1L isoform, similar to the IDI2 enzyme, is targeted to mitochondria (Cunillera *et al*., 1997). Again, single *fps* mutants, similar to *idi* mutants, are viable and fully fertile, while a double mutant is lethal (Closa *et al*., 2010). However, in contrast to IDI isoforms, the FPS isoforms are expressed differently in tissues, with FPS2 expressed at high levels only in seeds (Keim *et al*., 2012). The reasons of *fps1 fps2* mutant lethality was studied in detail, and it was revealed that the embryo inside the seed halts growth at the octant/early globular stage of development (Closa *et al*., 2010) (Fig. 2). This observation might be explained by the fact that although neither of the genes is expressed inside the young embryo, both *FPS1* and *FPS2* are highly expressed in the nutritional tissue called the chalazal endosperm. Hence, the deficiency of the maternal supply of FPP to the embryo prevents the synthesis of some important metabolites inside it (Keim *et al*., 2012). This crucial molecule might be ubiquinone, as mutants in a solanesyl diphosphate synthase and solanesyl transferase are halted at the same stage of development (Okada *et al*., 2004). It is worth mentioning that the *fps1 fps2* pollen is nearly sterile (Closa *et al*., 2010). Interestingly, the pollen grains are formed correctly and the pollen tubes grow *in vitro* but are unable to transmit the mutated alleles through the male line (Closa *et al*., 2010). A similar phenotype was never observed in sterol biosynthesis mutants, but was reported for dolichol biosynthesis mutants *pprd2* and *evn* (Jozwiak *et al*., 2015; Lindner *et al*, 2015). In *Solanaceae*, FPS plays a role in pollen–pistil communication but is not required for pollen development or germination; instead, it is a main factor in *S*-RNase-independent unilateral incompatibility (Qin *et al*., 2018). This function of FPS supports the hypothesis that molecules missing in *fps1 fps2* pollen, as well as in *pprd2* and *evn* pollen of Arabidopsis, are dolichols. Dolichol serves as an indispensable factor in protein glycosylation and GPI anchor synthesis of proteins important for gamete recognition (Tsukamoto *et al.*, 2010; Figueiredo *et al*., 2024).

#### Polyprenyl chain elongation

The FPP molecule is elongated further by the addition of consecutive isoprenoid modules, IPP molecules, and formation of the double C=C bond in *cis*(*Z*)-configuration, and a polyprenyl diphosphate is formed (Grabińska *et al*., 2016) (Fig. 3). In animals and fungi, solely the MVA pathway serves as a donor for both the *trans*(*E*)-precursor and isoprenoid moieties for elongation of the growing polymer chain. In plants, however, the biosynthetic origin of the dolichols remains under dispute (Fig. 1). It has been shown in a detailed study using inhibitors of MVA and MEP pathways and differently labeled precursor feeding followed by NMR and MS analysis that dolichols are mosaic compounds, built by MVA- and MEP-derived fragments (Jozwiak *et al*., 2017; Lipko *et al*., 2023). Nevertheless, the role of the MVA pathway in gametophytes and early embryos is predominant, as they do not have developed chloroplasts or active photosynthesis.

#### *cis*-Prenyltransferases—grab, link, and repeat

Enzymes responsible for the elongation of *all-trans*(*E*)-allylic precursor with IPP to form di- or tri-*trans*(*E*)-n-*cis*(*Z*) polyprenol chain are called *cis*-prenyltransferases (CPTs). These enzymes are catalytically active exclusively in the form of homodimers or heterodimeric complexes with accessory proteins (Grabińska *et al*., 2016). Plants possess several tissue-specific CPTs from both homo- and heterodimeric groups (Surowiecki *et al*., 2019). The majority of these enzymes are localized on the membrane of the endoplasmic reticulum (ER) but are also present in ER-derived structures such as lipid bodies (as is shown for yeast; Currie *et al*., 2014; Hoffmann *et al*., 2017) or lily anther tapetosomes (Liu *et al*., 2011) and *Taraxacum* rubber particles (Tan *et al*., 2023). Certain homomeric CPTs are found within chloroplasts (e.g. CPT7 in Arabidopsis, SlCPT5 in tomato), but their polyprenol products are not converted into dolichols (Akhtar *et al*., 2017). The ER/cytoplasmic and chloroplastic CPTs exhibit differences in their catalytic mechanisms (summarized in Grabińska *et al*., 2016). The former have a higher affinity for FPP than for GGPP. Additionally, most cytoplasmic/ER CPTs are heteromeric (except CPT1 in Arabidopsis) (Surowiecki *et al*., 2019), and their activity requires an accessory protein, such as LEW1 in Arabidopsis or SlCPT-BP in tomato (Brasher *et al*., 2015; Gawarecka *et al*., 2022) (Fig. 3). These accessory proteins may be directly engaged in the catalysis or may provide the anchorage to the ER membranes (Brasher *et al*., 2015; Edani *et al*., 2020). The anchoring of soluble CPT to the ER membrane enables the elongation of the polyprenyl chain simultaneously with its insertion into the lipid bilayer. Eukaryotic CPT enzymes usually produce a family of polyprenols with products differing in length by five carbon isoprenoid units (e.g. dolichol 15–17 with dolichol 16, i.e. composed of 16 isoprene units dominating in the case of CPT3). ER-produced polyprenols are further converted to dolichols (Jozwiak *et al*., 2015; Gawarecka *et al*., 2022).

There are seven ER/cytoplasmic CPTs encoded in the Arabidopsis genome (Table 1), with only some of them characterized. The housekeeping and ubiquitously expressed enzyme of this group is CPT3 (Gawarecka *et al*., 2022). This enzyme produces isoprenoid chains composed of 15–17 isoprene subunits, with polyprenyl-16 diphosphate dominant. The length and physiological roles of polyprenols produced by other ER/cytoplasmic CPT enzymes from Arabidopsis are unknown. Exclusively in flowers, precisely in tapetum and microspores of anthers, LLA66 *cis*-prenyltransferase of *Lilium longiflorum* is expressed (Liu *et al*., 2011; Yao *et al*., 2019), and the pattern of expression of this enzyme suggests a role in pollen coat formation.

#### Mutants of *CPT* genes

In Arabidopsis, *CPT3* is a housekeeping gene responsible for dolichyl phosphate biosynthesis in the whole sporophyte (Gawarecka *et al*., 2022). No changes in the sporophyte growth or fertility of the *CPT3*-RNAi or *CPT3*-overexpressing lines have been noted, while a significant reduction of the dolichol 15–16–17 content is observed. The development of gametophytes or seeds in this mutant has not been studied (Gawarecka *et al*., 2022) but, judging from very strong expression during seed development, it may be affected (eFP at https://bar.utoronto.ca). CPT3 co-localizes and interacts functionally with the LEW1 accessory protein at the ER (Gawarecka *et al*., 2022).

*CPT4* and *CPT5* are transcribed only in flower organs, in particular in pollen (Surmacz and Swiezewska, 2011; eFP at https://bar.utoronto.ca), but the phenotypic characterization of the mutants awaits detailed analysis. Based on phylogenetic analysis of protein sequences, CPT4 and CPT5 belong to the same family of cytosolic heterodimeric prenyltransferases forming dimers with the LEW1 accessory protein (Surowiecki *et al*., 2019). They do not have a specific C-terminal RXG motif, thus requiring interaction with LEW1, which contains this motif (Grabińska *et al*., 2017; Surowiecki *et al*., 2019; Edani *et al*., 2020). The architecture of heterodimeric *cis*(*Z*)-prenyl transferases producing dolichols (and natural rubber molecules) is preserved in plants such as *Taraxacum kok-saghys* (Niephaus *et al*., 2019; Muller *et al*., 2025) and tomato (Kutsukawa *et al*., 2022).

Other CPT enzymes present in the ER/cytoplasm of Arabidopsis, belonging to a homodimeric class, form a family of five members: CPT1, CPT2, CPT6, CPT8, and CPT9. From this class of enzymes, only CPT6 and CPT1 have been characterized (Surmacz *et al*., 2014; Surowiecki *et al*., 2019). CPT6 produces, quite unusually, a single, short-chain polyprenol (prenol 7; C35 long) and plays a role in the root cell response to abiotic stress (Kera *et al*., 2012), but it is not involved in protein glycosylation (at least upon heterologous expression in yeast) (Surmacz *et al*., 2014). *CPT6* expression is relatively high throughout seed development, reaching a peak at the mature seed (eFP browser at https://bar.utoronto.ca).

CPT1 produces long-chain polyprenols (18–23 isoprene units, C90–115 long) and its knockout is probably detrimental to plant survival since homozygous plants cannot be isolated (Surowiecki *et al*., 2019; Zhang *et al*., 2008 described the T-DNA mutant in *CPT1* that was erroneously ascribed to the *LEW1* gene at that moment, Fig. 3). *CPT1* is highly expressed in the root (Surowiecki *et al*., 2019), but also in the embryo during the heart stage of development, similar to *CPT2* (eFP browser at https://bar.utoronto.ca). CPT1 is involved in protein glycosylation in a complementation assay in yeast; such a role in plant cells awaits confirmation (Surowiecki *et al*., 2019). No work has been presented on CPT8 or CPT9. Interestingly, *CPT8* is almost solely expressed in the pistil and ovaries until the early post-fertilization stages (octant stage of the embryo) (eFP at https://bar.utoronto.ca), while *CPT9* is expressed in the root.

#### CPT-binding proteins—a helping hand in polyprenol synthesis

There is only one gene coding for the CPT enzyme accessory protein, *LEW1* in Arabidopsis, *SlCPTBP* in tomato (Brasher *et al*., 2015), and two genes in *T. kok-saghyz* (Niephaus *et al*., 2019). *lew1* mutants in Arabidopsis have been described (Zhang *et al*., 2008). The point mutation in the *LEW1* gene (initially assigned the CPT function) results in significantly decreased dolichol content, profound disorders of protein glycosylation in sporophytic tissues, electrolyte leakage through the membranes, and decreased resistance to drought (Zhang *et al*., 2008). The knockout of the *LEW1* gene by T-DNA insertion is non-viable; however, clearer characteristics of the reasons behind the lethality of *lew1* are lacking (Fig. 2). At least one of the gametophytes must be non-lethal because it is possible to obtain the heterozygous plant carrying one copy of point-mutated *LEW1* and one null copy (Zhang *et al*., 2008). *LEW1* is expressed in the flower tissues, but not in the embryo during seed development (eFP at https://bar.utoronto.ca).

LEW1 is a homolog of the NogoB receptor (NgBR) in mammals, which interacts with the human CPT enzyme (Harrison *et al*., 2011; Brasher *et al*., 2015; Grabińska *et al*., 2017). NgBR protein, in contrast to soluble CPT, is an intrinsic membrane protein and, in mammals, interacts with reticulon proteins (NgB, reticulon 4), shaping the ER (Jozsef *et al*., 2014). siRNA depletion of NgBR or genetic loss of NgBR in human cell lines has shown impaired sterol sensing and elevations in free cellular cholesterol because NgBR interacts with sterol-transporting NPC2 protein (Harrison *et al*., 2009; Park *et al*., 2014). These interactions of LEW1 with reticulons and sterol-binding proteins are not known in plants; however, if they exist, they may provide a regulatory role for cellular homeostasis and feedback interaction with the sterol-producing branch of the isoprenoid pathway, and also shed light on how dolichol biosynthesis (enzymes and products) influences ER membrane morphology and physical properties.

#### A long and winding road from polyprenol to dolichol

Once the di-*trans*(*E*)-n-*cis*(*Z*) polyprenyl diphosphate is synthesized by ER/cytosolic *cis*-prenyltransferase, it undergoes dephosphorylation by an as yet unidentified phosphatase and a consecutive reduction of the double bond in the isoprene unit closest to the hydroxyl group, catalyzed by polyprenol reductase (PPRD) belonging to the family of 5α-steroid reductases (Fig. 3). The respective mammalian enzyme is called SRD5A3 (Cantagrel *et al*., 2010). Recently, it has been suggested that polyprenol is first converted to an aldehyde polyprenal which undergoes hydrogenation to form dolichal by SRD5A3 and finally is converted back to alcohol—dolichol; both are alcohol/aldehyde oxidation/reduction reactions catalyzed by DHRXS enzyme as discussed in the following paragraphs (Wilson *et al*., 2024) (Fig. 3). The reduction step from polyprenol to dolichol (or polyprenal to dolichal) catalyzed by PPRD2 in dolichyl phosphate biosynthesis determines the efficiency of the whole biochemical pathway in plants (van Gelder *et al*., 2021), as well as in yeast (Szkopińska *et al.*, 2006). Further phosphorylation of dolichol leads to dolichyl phosphate. This step is catalyzed by a transmembrane ER enzyme called dolichol kinase (DOK; also known as EVAN in Arabidopsis) (Kanehara *et al*., 2015; Lindner *et al*., 2015) (Fig. 3).

#### Mutants in *PPRD2*

The step of double bond reduction in the polyprenol (-al) chain is characteristic only for isoprenoids inserted in the ER and other cytoplasmic membranes of eukaryotes. Polypreno(a)l reductase in Arabidopsis is encoded by two related genes (Jozwiak *et al*., 2015), and in tomato is encoded by only one gene (van Gelder *et al*., 2021). The *PPRD2* gene is expressed in all plant organs, while the *PPRD1* gene is only expressed in roots and flowers (Jozwiak *et al*., 2015). PPRD1 activity is not related to dolichol, but rather brassinosteroid synthesis (Wu *et al*., 2025). Detailed genetic analysis showed that *pprd2* T-DNA mutant pollen is sterile, while the female gametophyte transmission is normal (Jozwiak *et al*., 2015) (Fig. 2). Flower development (and possibly gametophytes, judging from dwarfed stamens and a much-reduced seed set) is strongly dependent on PPRD2 presence in the sporophytic tissue of the plant and cannot be substituted by PPRD1 (Jozwiak *et al*., 2015). The sterility of the *pprd2* pollen comes from the serious defects in pollen tube growth, such as swelling and branching, that may be rescued by dolichol supplementation to the *in vitro* germination medium. The same kind of deformations of pollen tubes can be seen when wild-type pollen germinates in the presence of the *N*-glycosylation inhibitor tunicamycin. Possible cell wall and exine deformations may also account for the decrease in the male gametophyte transmission rate; however, no pollen lethality is detected at the mature stage of the flower (Jozwiak *et al*., 2015).

**Fig. 4.**
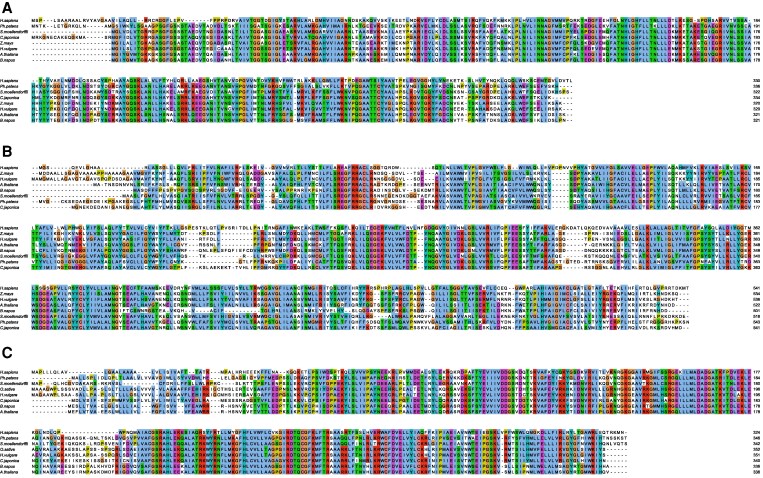
Phylogenetic analysis of genes putatively involved in isoprenoid metabolism in plants. (A) Homologs of the human *DHRSX* gene, (B) homologs of the human *RFT1* gene, and (C) homologs of the human *ALG5* gene. Phylogenetic analyses were performed using the most similar protein sequences from: *Homo sapiens* (NCBI), *Arabidopsis thaliana* (TAIR10), *Oryza sativa* (IRGSP-1.0), *Selaginella moellendorffii* (v1.0), *Physcomitrium patens* (Phypa v3.0), *Zea mays* (Zm-B73-REFERENCE-NAM-5.0), *Brassica napus* (AST_PRJEB5043 v1.0), *Hordeum vulgare* (Morex v3.0), and *Cryptomeria japonica* (NCBI v10.1). Amino acid sequences were extracted from the Ensembl Plants Database and NCBI database. Multiple sequence alignments were performed using Clustal Omega and visualized using Jalview v 2.0.

Recent findings in yeasts and humans suggest that the reduction of polyprenol to dolichol may be more complicated. It is performed in three enzymatic steps (Kentache *et al*., 2024; Wilson *et al*., 2024) (Fig. 3). In this case, the polyprenol molecule must be first converted to an aldehyde, polyprenal, formed by a DHRSX enzyme with the use of the NAD^+^ cofactor, then SRD5A3 (homologous to plant PPRD) reduces the C=C double bond in the α-terminal isoprene unit to form dolichal, and finally, again the DHRSX enzyme, this time acting as an NADPH-dependent reductase, catalyzes dolichol formation from the dolichal molecule (Wilson *et al*., 2024) (Fig. 3). Whether this double enzymatic function of DHRSX is present in plants has not been confirmed, but seems likely, since a highly homologous gene is present in analyzed plant species from different evolutionary groups (Fig. 4), in particular in Arabidopsis at locus At2g37540.

#### Mutants in *DOK*

The final step of dolichyl phosphate formation is phosphorylation catalyzed by DOK. This activity is encoded by a single gene, *DOK1* (known also as *EVAN*, *EVN*), in Arabidopsis (Kanehara *et al*., 2015; Lindner *et al*., 2015) (Fig. 3). Similar to the *pprd2* mutant, it is not possible to obtain homozygous *dok1* plants, although the sporophytes develop normally. Nearly half of the pollen from heterozygous *dok1* plants (T-DNA insertion mutants) is non-viable at anthesis and shows aberrant hollow morphology under SEM analysis (Kanehara *et al*., 2015), which has been also confirmed by an independent study through detailed genetic analysis of segregation in both T-DNA insertion and point mutation lines (Lindner *et al*., 2015) (Fig. 2). Consistent with this observation, the strong expression from the *DOK* promoter has been revealed in the anthers at developmental stage 8–9 of the flower (Kanehara *et al*., 2015), corresponding to early tricellular pollen (Lindner *et al*., 2015). Shortened siliques in *dok1* heterozygotes also suggest female gametophyte failure, and indeed many ovules are disorganized and remain underdeveloped (Kanehara *et al*., 2015) (Fig. 2). Parallel investigation has revealed that most female gametophytes in *evn* point mutants are correctly developed and able to attract and receive pollen tubes, but ∼25% of them do not undergo fertilization (Lindner *et al*., 2015). In most *evn* ovules, pollen tubes enter the synergid but are unable to stop growing and rupture; hence, the egg cells remain unfertilized, and instead large callose deposits are observed at the ovule entrance (Lindner *et al*., 2015). *DOK* RNAi-silenced lines are not affected; probably even the low amount of DOK sustains ovule fertilization (Lindner *et al*., 2015). Leaky knockdown mutants of *DOK1* flower early without any remarkable growth defect in vegetative tissues (Cho *et al*., 2017). Altogether, the effects of mutations in *PPRD2* and *DOK1* suggest a shortage of some glycosylated or GPI-anchored protein responsible for pollen–ovule interaction.

### *N*-Glycosylation of proteins—protein to be or not to be

*N*-Glycan (GlcNAc2Man9Glc3 tetradecaoligosaccharide) synthesis in the ER is relatively well conserved in eukaryotes. *N*-Glycan modification of polypeptides is crucial for protein folding and ER protein quality control, intracellular vesicular protein transport, and ER-associated degradation (ERAD). Correct folding and glycosylation permit protein oligomer formation and transport of proteins to their proper destination; incorrect folding or glycosylation targets the protein for degradation by the quality control system in the ER (J. Chen *et al*., 2024). Accumulation of the misfolded or incorrectly glycosylated proteins induces the unfolded protein response (UPR) in the ER. In humans, mutations in enzymes involved in both dolichol synthesis and the formation of dolichyl phosphate-linked oligosaccharides cause lethal congenital disorders of glycosylation with manifestations mainly in the nervous and immunological systems (Cantagrel and Lefeber, 2011; Buczkowska *et al*., 2015; Quelhas and Jaeken, 2024). The range of phenotypes for *N*-glycosylation mutants in plants is wide, from embryo lethality, through retarded growth and infertility, to salt sensitivity, most of which are probably related to protein quality control and the UPR (reviewed in Strasser *et al*., 2021; see Fig. 2).

#### Formation of glycan on the dolichyl phosphate precursor

**Fig. 5.**
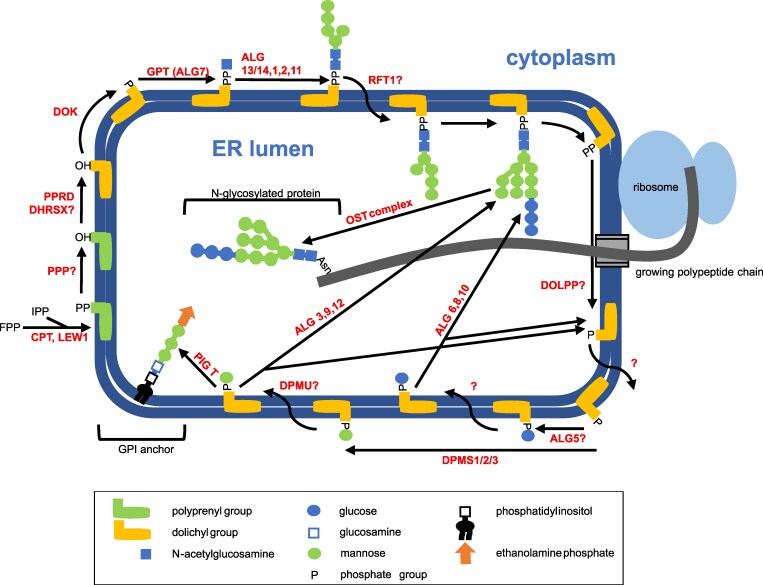
Schematic representation of dolichol-mediated glycosylation processes in plants. *N*-Glycosylation of proteins and synthesis of the GPI anchor in the endoplasmic reticulum. Names of the experimentally confirmed enzymes are given in bold, and putative steps are depicted with question marks. CPT, *cis*-prenyltransferase; LEW1, accesory protein of CPT; PPP, polyprenol diphosphate phosphatase; PPRD, polyprenal reductase; DHRSX, dehydrogenase/reductase (SRD family); DOK, dolichol kinase; GPT, GlcNAc-1-phosphotransferase; ALG13/14, UDP-*N*-acetylglucosamine transferase; ALG1, chitobiosyldiphosphodolichol β-mannosyltransferase; ALG2, α-1,3 mannosyltransferase; ALG11, α-1,2-mannosyltransferase; RFT1, dolichol scramblase; ALG3, α-1,3-mannosyltransferase; ALG9, α-1,2-mannosyltransferase; ALG12, α-1,6-mannosyltransferase; ALG6, α-1,3-glucosyltransferase; ALG8, α-1,3-glucosyltransferase; ALG10, α-1,2-glucosyltransferase; OST, oligosaccharyltransferase complex; DOLPP, dolichyl diphosphate phosphatase; ALG5, dolichyl-phosphate β-glucose synthase; DPMS1/2/3, dolichyl phosphate mannose synthase complex; DPMU, dolichyl phosphate mannose flippase; PIGT, glycosylphosphatidylinositol anchor (GPI anchor) transamidase; FPP, farnesyl diphosphate; IPP, isopentenyl diphosphate. Graphical symbols are explained on the scheme.

In *N*-glycosylation, the lipid-linked oligosaccharide precursor is assembled in a stepwise manner (Figs 3, 5) by Asn-linked glycosylation (ALG) enzymes on a dolichyl diphosphate (in detail, the synthesis of *N*-glycosylated protein and its transbilayer movement for non-plant eukaryotes is reviewed in Cantagrel and Lefeber, 2011; Rush, 2016). Generally, the eukaryotic pathways follow several steps. First, a dolichyl phosphate is used by the UDP-*N*-acetylglucosamine:dolichyl phosphate *N*-acetylglucosamine-1-P transferase (GPT; also known as GDPAT1) to start the assembly of the lipid-linked oligosaccharide (LLO) on the cytoplasmic face of the ER, with UDP-GlcNAc serving as a sugar donor. The product dolichyl diphosphate GlcNAc (Dol-PP-GlcNAc) is elongated to Dol-PP-GlcNAc2Man5 by enzymes using phosphonucleotide-activated sugars and then flipped to the lumen of the ER by a specific flippase RFT1 (S. Chen *et al*., 2024; Hirata *et al*., 2024). In parallel, dolichyl phosphate mannose (Dol-P-Man) is formed (from GDP-mannose) on the cytosolic side of the ER membrane by an enzymatic complex DPM1/DPM2/DPM3. The Dol-P-Man is flipped inside the ER lumen, where it serves as a sugar donor for LLO synthesis by enzymes ALG3, ALG12, and ALG9. Similarly, dolichyl phosphate glucose (Dol-P-Glc) is formed in the cytosolic side of the ER by the enzyme ALG5, with UDP-glucose serving as sugar donor and flipped inside the ER, where it is used for completion of the LLO before transfer of the glycan onto nascent proteins by ALG6, ALG8, and ALG10. The multi-subunit oligosaccharyltransferase (OST) complex catalyzes the transfer of the assembled oligosaccharide from dolichyl diphosphate to the nascent polypeptide in the lumen of the ER (Strasser *et al*., 2021).

Summarizing, dolichols play three subroles in protein *N*-glycosylation: first initial oligosaccharide build-up on the cytoplasmic side of the ER is constructed on the dolichyl diphosphate molecule (phosphorylated GlcNAc from the sugar-nucleotide precursor is transferred on dolichyl phosphate), next the Dol-PP-GlcNAc2Man5 is *en bloc* flipped in the membrane to the ER lumen and the dolichyl moiety probably enables local membrane destabilization for the flippase (S. Chen *et al*., 2024). Finally in the ER lumen, the oligo sugar is further built up by addition of new sugar molecules from dolichyl phosphate-sugar precursors (Fig. 5). In contrast to *N*-glycosylation, the primary mechanism for *O*-glycosylation in plants is unique among eukaryotes and proceeds via attachment of a sugar moiety to the hydroxyl group of the imino acid hydroxyproline and, less commonly, to the hydroxyl group of serine. The sugar moieties from nucleotide-sugar precursors are directly attached to protein, without the involvement of dolichyl phosphates (Gomord *et al*., 2010; De Coninck *et al*., 2021; Strasser *et al*., 2021).

#### Mutants in cytosolic nucleotide-sugar:dolichyl-phosphate glycosyltransferase-encoding genes

The first step in the synthesis of glycan for protein *N*-glycosylation is the formation of a dolichyl diphosphate-linked intermediate (DolPP-GlcNAc), which is conserved in all eukaryotes (Figs 3, 5). GPT is an enzyme that catalyzes this initial step of the biosynthesis of dolichyl-linked oligosaccharides in plants (Koizumi *et al*., 1999). GPT is a target of the antibiotic tunicamycin. Transgenic Arabidopsis seedlings overexpressing GPT grow even in the presence of 1.0 μg ml^–1^ tunicamycin, which kills the wild-type plant (Koizumi *et al*., 1999). Surprisingly, no mutants in the *GPT* gene have been known in Arabidopsis.

Dol-P-Man is a sugar donor for a branched oligosaccharide precursor that later may be transferred to the aspartate residue of a protein in the process of *N*-glycosylation or to phosphatidylinositol-GlcNAc to form GPI (Maeda and Kinoshita, 2008) (Figs 3, 5). In animals, Dol-P-Man also serves as a donor for *O*-glycosylation and *C*-glycosylation of proteins, but these processes undergo different pathways in plants (Larsen *et al*., 2019; De Coninck *et al*., 2021). The formation of Dol-P-Man on the cytoplasmic side of the ER is catalyzed by the dolichol phosphate mannose synthase (DPMS) enzyme, which in yeast is a monomeric protein, but in animals and plants is a complex of three subunits encoded by *DPMS1*, *DPMS2*, and *DPMS3* genes (Maeda and Kinoshita, 2008; Jadid *et al*., 2011). Similar to *cis-*prenyl transferases, where LEW1 protein anchors the enzymatic complex to the ER membrane, also in the case of DPMS, the catalytic subunit, DPMS1, is devoid of the transmembrane domain and attached to the membrane by transient interactions with the other two subunits (Maeda and Kinoshita, 2008; Jadid *et al*., 2011). The DPM2 subunit, apart from being a part of the DPMS complex, also plays a role in the transfer of a mannose residue from Dol-P-Man on the GPI anchor mediated by the PIGT (GPI anchor transamidase) enzymatic complex (Maeda and Kinoshita, 2008) (Fig. 5). Arabidopsis plants carrying the T-DNA insertion in the *DPMS1* gene are viable, but weak and chlorotic. *dpms1* T-DNA insertion mutants and lines with *DPMS1* transcription reduced by RNAi are hypersensitive to increased ammonium concentration in the medium and affected by UPR stress (Jadid *et al*., 2011). Both *N*-glycosylation and GPI anchor formation are impaired in the mutants. *dpms* mutants form wrinkled seeds, indicative of cell wall malformation (Jadid *et al*., 2011); unfortunately, no detailed study on gametophytes has been performed.

These relatively mild phenotypes of *dpms1* mutants are surprising, taking into account that Arabidopsis plants carrying mutations in the upstream part of dolichyl phosphate synthesis (*PPRD2* and *DOK*) (Jozwiak *et al*., 2015; Kanehara *et al*., 2015; Lindner *et al*., 2015) as well as downstream GPI synthesis (Gillmor *et al*., 2005; Bundy *et al*., 2016) and *N*-glycosylation (Boisson *et al*., 2001; Gillmor *et al*., 2002; Lindner *et al*., 2015) are lethal or severely impaired (Fig. 2). The ion leakage through membranes and connected water loss phenotype, not caused by a stomatal opening defect, are common to *lew1* and *dpms* mutants (Zhang *et al*., 2008; Jadid *et al*., 2011) and may be explained by a role for dolichols and polyprenols in abiotic stress response (Bajda *et al*., 2009; Dmuchowski *et al*., 2021). If this protective effect of long-chain isoprenoids is achieved by their role as membrane modifiers (as suggested by *in vitro* model membrane studies: Haines, 2001), cofactors in cell wall biosynthesis, or protective waxy substances on the surface of leaves remain to be experimentally elucidated.

Another sugar precursor for *N*-glycosylation in the lumen of the ER is Dol-P-Glc (Figs 3, 5). In animals and yeast, it is synthesized at the cytoplasmic site of the ER by a membrane-attached enzyme ALG5 from dolichyl phosphate and UDP-glucose. In plants, the activity is present (glucans modified by glucose are synthesized in the ER), but the identity of the ALG5 homolog is unknown. Our phylogenetic analysis has shown that the protein homologous to ALG5 is encoded in Arabidopsis at locus At2g39630, and other plant genomes (Fig. 4).

#### Mutants in lumenal dolichyl-phosphate-sugar:dolichyl-diphosphate-oligo sugar glycosyltransferase-encoding genes

Dol-P-Man in the ER lumen is a donor of mannose residues for further synthesis of the dolichyl-linked oligosaccharide (Fig. 5). The additions of mannose are executed by the subsequent action of ALG3, ALG9, and ALG12 enzymes, all present in animals, fungi, and plants (Oriol *et al*., 2002) (Fig. 5). ALG3 functions in Arabidopsis are connected to pathogen recognition by glycosylated extracellular domains of pattern recognition receptors (Trempel *et al*., 2016). Interestingly, despite protein *N*-glycosylation defects, *alg3* grows as well as the wild type under normal and elevated temperature or salt/osmotic stress conditions that induce the UPR (Henquet *et al*., 2008; Kajiura *et al*., 2010). Also, *alg9* and *alg12* knockouts in Arabidopsis are viable. ALG9 and ALG12 are engaged in the UPR and degradation of misfolded extracellular receptors, for example BRI1 and FLS (Hong *et al*., 2012; Trempel *et al*., 2020).

Dol-P-Glc in the ER lumen donates the terminal glucose molecules to the growing dolichol-linked oligosaccharide (DLO) (Fig. 5). These modifications are achieved by the action of ALG6, ALG8, and ALG10 glycosyltransferases, conserved from yeast to animals and plants (Maeda and Kinoshita, 2008). The inactivation of *ALG10* in Arabidopsis results in altered leaf size, activation of the UPR, and increased salt sensitivity (Farid *et al*., 2011), while mutants in *ALG6* and *ALG8* genes of plants have not been described.

#### Mutants in dolichyl-diphospho-oligosaccharide:*N*-asparagine transferase complex- (OST) encoding genes

The dolichyl diphosphate-glycan is finally co-translationally transferred onto the asparagine moiety in a protein (Fig. 5). This process takes place in the ER lumen and is catalyzed by a large OST complex, consisting of nine proteins in eukaryotes (Strasser *et al*., 2021; Ramirez and Locher, 2023). In contrast to relatively mild phenotypes of deficiency of dolichyl phosphate-dependent mannosyl- and glycosyltransferases, knockouts of OST complex subunit-encoding genes are lethal in Arabidopsis. The null *dgl1-2* allele, which is impaired in *N*-glycosylation, is lethal at early stages of embryo development (Lerouxel *et al*., 2005) due to aberrant glycosylation of a cell wall polymer-synthesizing enzyme (Fig. 2). The glycosylation defect in this mutant is not accompanied by changes in the cellulose content. In contrast, the matrix polysaccharides in *dgl1-2* include an increased arabinose content, suggesting that pectin and/or arabinogalactan protein (AGP) biosynthesis is partially altered (Lerouxel *et al*., 2005); they also contain abnormal callose deposits. Another member of the OST complex is the SST protein. The *stt3a stt3b* double mutation is gametophytic lethal (Fig. 2), while *the sst3a* knockout is insensitive to increased salt concentrations (Koiwa *et al*., 2003). Glycosylation patterns of cell surface proteins may represent an important mechanism of gametophyte recognition. Mutation in another OST complex protein OST3/6-encoding gene (*ARTUMES*) causes pollen tube overgrowth and a failure to deliver the sperm cells in inter-species pollination due to lack of *N*-glycosylated protein recognition between the gametes (Muller *et al*., 2016) (Fig. 2). A mutant in another member of the OST complex has been found in a screen for aberrant pollen tube growth in the style (Johnson *et al*., 2004) (Fig. 2). In both cases, the phenotypes are reminiscent of *fps1 fps2*, *pprd2*, and *dok1* mutants in dolichyl phosphate synthesis. Mutants in *OST3/6* show aberrant glycosylation of the cell surface pathogen receptor and cellulose-synthase complex accessory protein KORRIGAN1, but not the BRI1 receptor (Farid *et al*., 2013). OST3/6 deficiency results in activation of the UPR and causes hypersensitivity to salt/osmotic stress (Farid *et al*., 2013).

To summarize, the defects in protein *N*-glycosylation vary from mild to gametophytic lethal and do not correspond to the order of reactions or subcellular localization of the processes catalyzed by the dolichyl phosphate-dependent enzymes. This issue raises the possibility either of the redundancy of enzymatic activities in Arabidopsis, which is not confirmed by evolutionary analysis, or that the enzymes (and proteins involved in the recognition of glycans) are not very specific towards their substrates. Also, some of the alleles analyzed so far are not knockouts, but ‘leaky’ knockdowns. The last possibility may be the case, as corresponding gene lesions in humans or other vertebrates cause very serious developmental defects leading to mortality (reviewed in Quelhas and Jaeken, 2024; Cantagrel and Lefeber, 2011).

### GPI-modified proteins—at the first line of recognition

GPI-anchored protein destination is the outer leaflet of the plasma membrane. In plants, GPI-modified proteins participate in cell wall deposition and remodeling, defense responses, and cell signaling (Hromadova *et al*., 2021; Yu *et al*., 2021; Zhou, 2022). More than half of Arabidopsis AGPs carry GPI modification (Leszczuk *et al*., 2023). The GPI anchor consists of saturated acyl chains inserted in the outer plasma membrane leaflet, phosphoinositol, and glycan molecules facing the exterior of the cell. GPI-anchored proteins are directed by their long saturated acyl chains to sterol-enriched microdomains, where they can serve in signaling functions or interact with certain enzymes (Levental *et al*., 2010). AGPs play a role in cell recognition during development, fertilization, or response to pathogens (Desnoyer and Palanivelu, 2020; Silva *et al*., 2020). For example, an Arabidopsis GPI-anchored protein, LORELEI, functions in pollen tube reception of female signals, double fertilization (Tsukamoto *et al.*, 2010), and early seed development, and COBRA-LIKE10 (COBL10) regulates the polar deposition of wall components in pollen tubes growing inside female tissues and is critical for micropylar guidance (Dai *et al*., 2014). JAGGER protein is essential for polytubey block (so no more than one pollen tube fertilizes the ovule) and synergid cell degeneration (Pereira *et al*., 2016; Figueiredo *et al*., 2024).

#### GPI anchor synthesis

GPI is synthesized in the ER membrane, where it binds to the C-terminus of the newly synthesized proteins (Fig. 5). The four mannoses of the GPI anchor are added in the ER lumen (Maeda *et al*., 2001). The first mannose is added by PIG-M, the second mannose is added by PIG-V, and the last two by PIG-B and SMP3 mannosyltransferases, respectively. All four enzymes use the same Dol-P-Man donor substrate and catalyze reactions similar to those of the corresponding asparagine-linked glycosylation (ALG) mannosyltransferases of the *N*-glycan series (Oriol *et al*., 2002). The PIG-B, PIG-M, and SMP3 mannosyltransferases have been identified in Arabidopsis by homology search (Oriol *et al*., 2002). These enzymes from distinct evolutionary groups share similar transmembrane motifs enabling dolichol recognition in the membrane bilayer (Zinkle *et al*., 2025). Mutations in PIG genes in Arabidopsis are severe in comparison with the DPMS complex mutation described in the preceding paragraphs (Gillmor *et al*., 2005; Jadid *et al*., 2011), and correspond more to the lethal defects presented in upstream dolichol synthesis genes such as *PPRD2* or *DOK1* (Jozwiak *et al*., 2015; Lindner *et al*., 2015) (Fig. 2).

*pnt1* (PIG-M) pollen is less viable than that of the wild type, and *pnt1* embryos are delayed in morphogenesis and show enlargement of the shoot, disorganization of root meristems, and multiple cotyledons or no cotyledons at all (Gillmor *et al*., 2005). *pnt1* embryos contain less crystalline cellulose, but more pectin; xyloglucan, pectin, and callose are ectopically distributed. Mutants in *PIG-M* embryos have negligible amounts of GPI-anchored proteins; probably, the absence of correct modification enhances protein degradation. The altered *pnt1* segregation ratio is due to poor transmission through pollen, but this trait has been studied only genetically (Gillmor *et al*., 2005).

Further mannosylations in GPI synthesis are performed by the PIG-B enzyme with Dol-P-Man as sugar donor. Interestingly, mutations in this gene in Arabidopsis cause pollen sterility and embryo lethality as well (Dai *et al*., 2014) (Fig. 2). No difference in viability or morphology has been detected between the *PIG-B* (*aptg1*) mutant and the wild-type mature pollen grains, but the pollen tube guidance *in vivo* is strongly impaired (Dai *et al*., 2014). The development of *aptg1* embryos is retarded at the globular embryo or earlier stages. Mutations in PIG-V or SMP3 in Arabidopsis have not been reported.

### Transmembrane transport and recycling of dolichyl phosphate and dolichyl-bound oligosaccharides

For Dol-P-Man, Dol-P-Glc, and Dol-PP-GlcNAc2Man5 to serve in GPI synthesis, they must be flipped inside the ER lumen (Fig. 5), and this step is supposedly achieved for Dol-P-Man by the MDPU protein in humans (Maeda and Kinoshita, 2008), but by an as yet unknown flippase in plants. The identity of the Dol-P-Glc flippase is not clear either in plants or in other eukaryotes. The flippase for Dol-PP-GlcNAc2Man5 has, after a long-term effort, been recently identified as RFT1 in humans and yeasts (J. Chen *et al*., 2024; Hirata *et al*., 2024). The whole process of *trans*-bilayer transport and recycling of dolichol-based intermediates awaits elucidation in plants, but a gene homologous to *RFT1* exists in the analyzed plant species (Fig. 4), in Arabidopsis represented by the locus At5g07630.

After donating the glycan molecule to the Asn residue in a protein by the OST complex, the dolichyl diphosphate molecule must be recycled to perform the glycosylation–cofactor function again. A dolichyl diphosphate phosphatase has been identified in yeast (CWH8) and humans (DOLPP1) (Fernandez *et al*., 2001; Rush *et al*., 2002), but has not been characterized in plants (Figs 3, 5). This enzyme is involved in the recycling of dolichol after oligosaccharide transfer to a protein, by removing one phosphate from dolichyl diphosphate in the ER (Figs 3, 5). Translocation of dolichyl phosphate from the ER lumen to the cytoplasmic side of the ER membrane is facilitated by a specific flippase in mammals (Rush *et al*., 2002), and the efficiency of this process plays a major role in the regulation of the dolichyl phosphate pool available for the glycosylation pathway (Schenk *et al*., 2001a).

Interestingly, the flippases for Dol-PP-GlcNAc2Man5, Dol-P-Man, and Dol-P-Glc are energy independent and catalyze an extremely rapid equilibration of DLO intermediates from the luminal to the cytosolic side of the ER membrane and in the reverse direction, which is in contrast to phospholipid flippases, which are energy-consuming, unidirectional enzymes (Rush, 2016). The biophysical perturbations that dolichol and/or dolichyl phosphate induce in the membrane may facilitate fast lipid scrambling, as discussed in the following paragraph.

### Dolichol as a membrane modifier

#### Between the leaflets—conformation of dolichol and its derivatives

**Fig. 6.**
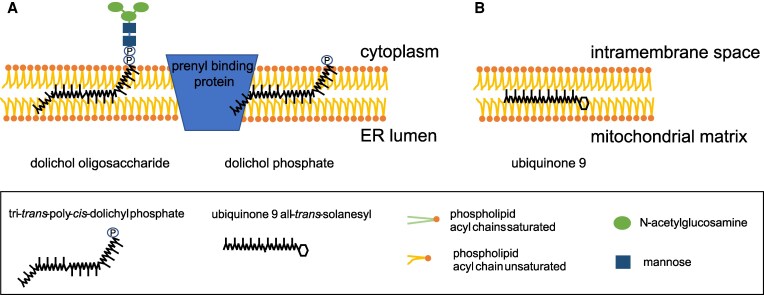
Schematic representation of the localization of long-chain linear isoprenoids in the biological membrane. Modes of prenyl group interaction with the bilayer are depicted **A)** for dolichol according to Hartley and Imperiali (2012) and **B)** for solanesol according to Quirk *et al*. (2016). The scale of the length of isoprenoid groups in comparison with fatty acid chains is preserved. Graphical symbols are explained on the scheme.

In animal tissues, and possibly in plants as well, dolichyl phosphate accounts for <10% of total dolichol content (Cantagrel and Lefeber, 2011). Up to 60% of dolichol is found in the form of fatty acid esters (most probably in storage form, accumulated in lipid droplets, similar to sterol esters), and the rest as free alcohol (Cantagrel and Lefeber, 2011). The biological role of free dolichol is less studied than that of dolichyl phosphate. Structural studies suggest that dolichol accepts helical conformation in the membrane due to a large number of *cis*(*Z*)-double bonds (Fig. 6). The size of such a molecule reaches ∼30 Å (Zhou and Troy, 2005). An explanation of dolichol conformation comes from molecular mechanics modeling methods and has been confirmed by small-angle X-ray scattering visualization. It has been discovered that instead of spanning through the bilayer, dolichol is localized between the leaflets of the membrane. According to this model, the solution conformation of dolichol is a central coiled region flanked by two opposing arms directed towards bilayer surfaces (Murgolo *et al*., 1989) (Fig. 6). In contrast, the phosphate head group of dolichyl phosphate is exposed at the bilayer surface, while the rest of the molecule is inserted in the bilayer at an angle (Zhou and Troy, 2005; discussed in detail in Hartley and Imperiali, 2012) (Fig. 6).

#### Flipping and scrambling of the membrane components

Some roles which dolichols play in the cell may be directly connected to their shape and include ER membrane organization—induction of the hexagonal phase II, but also induction of membrane fluidity and *trans*-bilayer lipid movement, as shown in *in vitro* biophysical experiments (Valtersson *et al*., 1985; van Duijn *et al*., 1986) (Fig. 6). In model membranes, dolichol, in contrast to cholesterol, does not affect the thermotropic behavior of membranes. Dolichol phosphate incorporation in the membrane abolishes the transition from the gel to the liquid crystalline phase (Valtersson *et al*., 1985). Dolichol phosphate increases the fatty acid movement and fluidity of the membranes; however, this effect is confined to the isoprenoid moiety with specific bond geometries, namely the presence of two or three double bonds in *trans*(*E*) conformation near the ω-end followed by several double bonds in *cis*(*Z*) conformation (Ciepichal *et al*., 2007; Entova *et al*., 2019). Dolichyl phosphate induces hexagonal phase II transition and may elicit the transmembrane movement (flip/flop) of glycosylated lipid intermediates, as has been suggested by Valtersson *et al*. (1985) (Fig. 6). Both dolichol and dolichyl phosphate enhance vesicle fusion in model membranes (van Duijn *et al*., 1986). Prenyl-recognizing peptides and proteins bind to the dolichol surface and reverse its non-bilayer-forming properties (Zhou and Troy, 2003, 2005). So far, all these observations have been delivered from biophysical experiments on artificial membranes built of a limited number of phospholipid species and devoid of proteins. Hypotheses that dolichols and dolichyl phosphates may *in vivo* locally influence membrane properties, acting as fusogenic and destabilizing agents, await experimental confirmation in an *in vivo* system.

#### Dolichol—a hydrophobic seal for the ion leakage?

An interesting hypothesis based on structural studies on model membranes enriched in isoprenoid lipids is that the branching of the hydrocarbon chains in the space crowds the hydrocarbon between the leaflets of the membrane and makes it more impermeable to proton leakage (Haines, 2001) (Fig. 6). Although based on theoretical divagations, nevertheless the theory may turn out to be true for biological membranes; the electrolyte leakage has been observed for plants with knockouts in the genes involved in dolichol synthesis and metabolism (Zhang *et al*., 2008; Jadid *et al*., 2011).

### Dolichol—an impregnation for the seeds, pollen, and other plant organs?

It has not been proven in plants yet, but it seems likely that dolichols (or their acyl esters) serve as extracellular protective waxy substances, similar to other hydrophobic molecules such as fatty acid esters, hydrocarbon alcohols, and sterol esters (Buschhaus and Jetter, 2011; Lewandowska *et al*., 2020; Garcia-Coronado *et al*., 2022). Mutants in the MVA pathway-encoding genes, upstream from dolichol, flaky pollen *fkp* (HMG synthase), and *hmgr1* show pronounced maternal sporophytic defects in pollen coat formation. The tapetosomes (derivatives of ER), specific lipid-containing organelles released by the tapetum onto the surface of developing pollen grain, are not correctly formed or deposited in these mutants (Suzuki *et al*., 2009; Ishiguro *et al*., 2010; Kobayashi *et al*., 2018). Also, it is worth mentioning that in *Saccharomyces cerevisiae*, long-chain dolichols not involved in protein glycosylation are produced by a separate *cis*-prenyltransferase Srt1 (Schenk *et al*., 2001b; Hoffmann *et al*, 2017; Chrissian *et al*., 2020), and this process is conserved in other fungi. These lipids play a role in the formation of a protective cell wall layer of the post-meiotic spore by forming lipid insulation and by inducing cell wall-synthesizing enzyme activity (Hoffmann *et al*., 2017). It may be speculated that dolichols in other plant organs, for example roots and seeds, may also be secreted on the surface and play protective roles, similar to triacylglycerols, sterol esters, acyl-derived waxes, and alkenes (K. Chen *et al*., 2024; Xu *et al*., 2024). For example, in Arabidopsis and other plants, an additional family of long-chain dolichols has been isolated solely from the roots and flowers (anthers)—organs excreting lipid substances on the surface (Jozwiak *et al*., 2013; Surowiecki *et al*., 2019; Lipko *et al*., 2023)—and some long-chain dolichol-synthesizing plant CPT enzymes are not involved in protein glycosylation, but serve another, as yet unidentified function. The insulation hypothesis awaits experimental confirmation through detailed studies of plants grown in natural environmental conditions, followed by isolation and identification of extracellular deposited lipids.

## Ubiquinone

### Ubiquinone functions

**Fig. 7.**
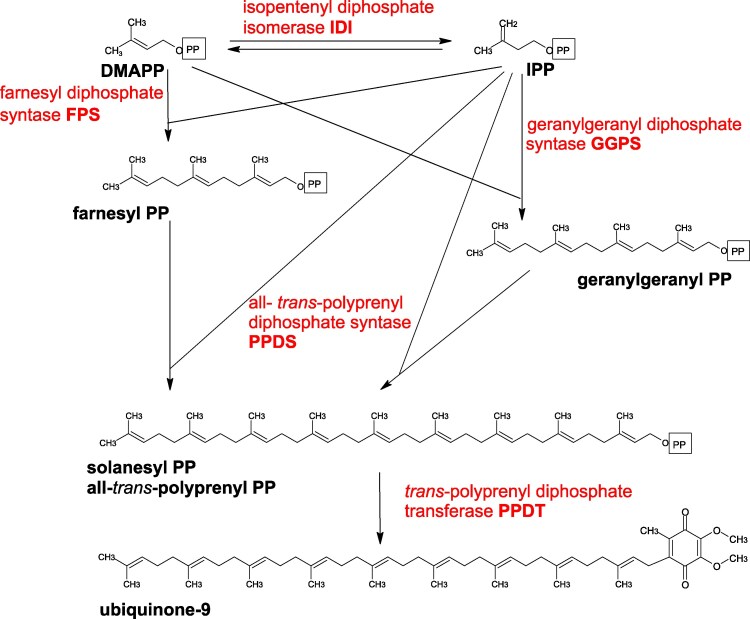
Biochemical pathways involved in ubiquinone synthesis. For clarity, only isoprenoids and related metabolites are shown with full chemical formulae. Full names of the enzymes and EC numbers are summarized in Table 1. Picture prepared in ChemSketch.

In eukaryotes, ubiquinone is a central component of mitochondrial oxidative phosphorylation, mediating the electron transfer from complex I (NADH:ubiquinone oxidoreductase) and II (succinate dehydrogenase) to complex III (cytochrome *bc*1 oxidoreductase). Ubiquinol (reduced ubiquinone) is also a lipid-soluble antioxidant in all cellular compartments (recent advances in understanding ubiquinone synthesis and function in plants are presented in Xu *et al*., 2022). The ubiquinone molecule is built of a benzoquinone ring modified by additional methylation and methoxylation and a prenyl anchor (Fig. 7). In plants, the dominating lipid moiety in ubiquinone is 9-isoprene units long (45 carbon) *all-trans*(*E*)-solanesol (dominating in Arabidopsis and rice) or 10-isoprene units long *all-trans*(*E*)-spadicol (dominating in tomato and soybean) (Xu *et al*., 2022).

### Ubiquinone prenyl chain in the membrane—similar to, yet different from, dolichol

Prenylated ubiquinone resides in the mitochondrial inner membrane with at least part of the isoprenyl side chain embedded within the bilayer midplane, and the headgroup (ring structure) directed towards one of the membrane phospholipid group surfaces or hidden in the bilayer plane (Quirk *et al*., 2016) (Fig. 6). The prenyl group of ubiquinone in Arabidopsis is 30 carbon atoms shorter than that of the dolichol chain, but all double bonds are in the *trans*(*E*)-conformation, meaning that the overall shape of the molecule must be more elongated and regular than in case of the mixed poly-*trans*(*E*)–poly-*cis*(*Z*) structure of the dolichol chain. It is believed that the ubiquinone prenyl group is present in the membranes, not in the fully extended conformation spanning through the whole bilayer diameter, but bent or perpendicular to the acyl chains of lipids (Galassi and Arantes, 2015; Kaurola *et al*., 2016). Similar to dolichol, this means that the prenyl chain of ubiquinone is hidden between the acyl chains. The results from molecular dynamics modeling (Galassi and Arantes, 2015; Kaurola *et al*., 2016) have been confirmed by biophysical experiments on model membranes (Quirk *et al*., 2016; Braasch-Turi *et al*., 2022).

### Ubiquinone prenyl chain biosynthesis: a controversial case

Initial studies in plants identified the location of ubiquinone prenyl chain biosynthesis as ER/microsome membranes (Swiezewska *et al*., 1993). Then, for a long time, it was believed that mitochondria possess their own system of solanesol biosynthesis consisting of the enzyme producing FPP precursor (FPS1L, Closa *et al*., 2010) and/or geranylgeranyl precursor (GGPS1) and *trans*(*E*)-polyprenol synthase itself (reviewed in Kopcsayova and Vranova, 2019); however, the functionality of mitochondrion-targeted geranylgeranyl diphosphate (GGPP) synthase (GGPS1) is questionable (Ruiz-Sola *et al*., 2016a). The identity of the enzyme synthesizing solanesyl (and spadicyl) diphosphate in plants has also been the subject of a long dispute (Bouvier *et al*., 2000; Hirooka *et al*., 2003; Jun *et al*., 2004). Finally, it has been established that the enzyme responsible for the synthesis of *all-trans*(*E*)-prenyl diphosphates is PPDS double localized to mitochondria and chloroplasts (Ducluzeau *et al*., 2012) (Figs 1, 7).

#### Synthesis of geranylgeranyl diphosphate in mitochondria—still an open question

In plants, the main site of GGPP synthesis is the plastid, where the IPP and DMAPP precursors are derived exclusively from the MEP pathway (Wang *et al.*, 2023), directly or by IPP isomerization catalyzed by the IDI1 enzyme (Phillips *et al*., 2008). GGPP in plastids is metabolized further to photosynthetic pigments: chlorophyll side chains, carotenoids, and other important chloroplast membrane constituents such as plastoquinone, phylloquinone, tocopherols, or long-chain polyprenols (Ruiz-Sola *et al*., 2016b; Akhtar *et al*., 2017; Lipko *et al*., 2023; see Fig. 1) Similarly to FPP, some pool of GGPP is also synthesized in the plant cell cytoplasm, mainly by the GGPS11S isoform of the GGPP11 synthase (Ruiz-Sola *et al.*, 2016a, b). The GGPS enzyme catalyzes the iterative elongation of DMAPP allylic precursor with IPP; all three added isoprene units are in the *trans*(*E*) conformation. In the cytoplasm, IPP and DMAPP come from the cytoplasmic pool produced by short isoforms of the enzymes IDI1 and IDI2 (Phillips *et al.*, 2008; Sapir-Mir *et al*., 2008). The GGPS11S isoform, similarly to IDI1S and IDI2S isoforms, is a product of translational regulation of the gene *GGPS11* encoding both organellar (long) and cytoplasmic (short) isoforms (Phillips *et al*., 2008; Sapir-Mir *et al*., 2008; Ruiz-Sola *et al*., 2016a). Plants devoid of the GGPS11S isoform are lethal, and it was proposed, that the metabolite missing in embryo development might be ubiquinone (Ruiz-Sola *et al*., 2016a) (Fig. 2). Additionally a product of another GGPP synthase-encoding gene *GGPS1* is targeted to mitochondria, where it may serve for the synthesis of GGPP for solanesyl (or spadicyl) diphosphate synthesis *in situ.* In that case, the precursors, IPP and DMAPP would come from the mitochodrial pool synthesized by the IDI2L isoform *in situ* or from the pool imported from the cytoplasm (Fig. 1). Some import of the GGPP itself from the cytoplasm (coming from GGPS11S enzyme action) or a switch for FPP in the case of GGPP shortage must be considered since *ggps1* mutants are viable, in contrast to *ggps11* and *ppt1* mutants downstream in ubiquinone synthesis described in the following paragraphs (Okada *et al*., 2004; Ruiz-Sola *et al*., 2016b) (Figs 1, 2). The mechanism of GGPP transport from the cytoplasm to mitochondria, as well as GGPP transport from the chloroplasts to cytoplasm remains unknown.

#### Synthesis of *all-trans*(*E*)-prenyl diphosphates

Solanesyl diphosphate (SPP) is synthesized in mitochondria by an enzyme called solanesyl diphosphate synthase (or *trans*-polyprenyl diphosphate synthase; it is also the source of spadicyl diphosphate) (Ducluzeau *et al*., 2012). The homo-dimeric enzyme catalyzes the head-to-tail iterative elongation of *trans*(*E*)-GGPP or *trans*(*E*)-FPP with IPP, introducing all-new isoprenoid modules in *trans*(*E*)-configuration, in contrast to *cis*(*Z*)-prenyltransferases described in preceding paragraphs (compare Fig. 7 with Fig. 3). The biosynthetic routes and mutants in Arabidopsis isopentenyl diphosphate isomerase and farnesol diphosphate syntase are described at the beginning of the section considering dolichol synthesis; it should be reiterated here that IPP and FPP are synthesized in mitochondria by IDI2L and FPS1L isoforms, respectively (Cunillera *et al*., 1997; Phillips *et al*., 2008; Sapir-Mir *et al*., 2008; Keim *et al*., 2012), or may be imported from the cytoplasmic pools. The unclear origin of GGPP is discussed in the preceding paragraph.

The *cis*(*Z*)-prenyltransferases (producing dolichol precursors) and *trans*(*E*)-prenyltransferases (producing solanesol) belong to two different, neither evolutionarily nor structurally related families (Kopcsayova and Vranova, 2019). *In vitro*, the enzyme PPDS accepts equally well GPP (geranyl diphosphate), FPP, and GGPP, and releases a family of products ranging in length from five to nine isoprene units, with seven isoprene units being the most abundant (Hsieh *et al*., 2011), so its *in vivo* specificity for the length of the product is somehow different (nine or 10 isoprene units). Similar mitochondrial SPPS activity was also described in rice (Ohara *et al*., 2010).

The Arabidopsis *ppds* RNAi lines retain 20% of the ubiquinone pool, but no changes in plastoquinone amount were noted (Ducluzeau *et al*., 2012). In the T-DNA line in the *PPDS* gene, 20% embryo lethality (or female gametophyte lethality) is observed; however, detailed genetic analysis or microscopic observations are missing (van Schie *et al*., 2007) (Fig. 2).

#### Transfer of the *all-trans*(*E*)-prenyl group to the precursor of the benzoquinone ring

The solanesyl (or spadicyl) diphosphate molecule is transferred to the 4-hydroxybenzoate (precursor of the benzoquinone ring) on the mitochondrial membrane by an enzyme called 4-hydroxybenzoate:polyprenyl diphosphate transferase (polyprenyl transferase PPT1; Fig. 7). In *in vitro* experiments, the Arabidopsis enzyme accepts a wide range of substrates, ranging from 10-carbon-long GPP to 45-carbon-long SPP (Okada *et al*., 2004). A regulatory mechanism of co-expression of synthesis of both the benzoquinone ring and prenyl tail of ubiquinone exists in eukaryotes, including plants, that ensures an equal amount of both precursors of the quinone ring and prenyl tail for ubiquinone synthesis (Ducluzeau *et al*., 2012; Hayashi *et al*., 2014). The transfer of the lipid group to 4-hydroxybenzoate is a rate-limiting step in ubiquinone synthesis in plants, such as *Salvia mimiltiorrhiza*, tobacco, and tomato (Ohara *et al*., 2004; Liu *et al*., 2019; Fan *et al*., 2021). Plants with increased levels of ubiquinone due to overproduction of biosynthetic enzymes are more resistant to abiotic stresses, mainly due to improved radical scavenging ability (Ohara *et al*., 2004; Liu *et al*., 2019).

The T-DNA insertion in the gene encoding *PPT1* in Arabidopsis results in embryo lethality at the transition from the globular to the heart stage of development (Okada *et al*., 2004) (Fig. 2). Pollen carrying the mutated allele is fertile, but unfortunately no details on allele transmission have been presented (Okada *et al*., 2004). These results are in line with the observed phenotypes of other Arabidopsis mutants in ubiquinone ring biosynthesis pathway genes, which are also embryo lethal at the late globular stage of embryogenesis (Latimer *et al*., 2021; Hu *et al*., 2023).

## Perspectives

Long-chain linear isoprenoids are not abundant lipids in the eukaryotic cell, yet they play crucial roles in basic metabolism. Knowledge about their functions is limited in plants compared with mammals or yeast, but it has emerged as an important field of study where membrane physics meets cell physiology. So far, most experiments on dolichol and solanesol (and their derivatives) localization, conformation, and interaction with other lipids and proteins have been performed in artificial membranes due to the technical limitations of biophysical and microscopic techniques. New and improved visualization methods may lead to discoveries of long-chain isoprenoid biophysical properties in living cells. In recent years, progress has also been made on the biochemistry of dolichol and solanesol (spadicol) turnover in yeast and humans; still, many enzymatic activities remain undescribed in plants. Only a few enzymatic steps in dolichol synthesis, transport, and degradation have gene annotations, and even fewer have been addressed experimentally. The particularly elaborated isoprenoid metabolism in plants, with separate yet interchangeable pools of precursors that are regulated by internal and environmental cues, awaits detailed metabolomic description and flux modeling. From a biotechnological and industrial point of view, biofortification strategies for the ubiquinone build-up in plant-derived foods have only recently achieved success. Additionally, last but not least, the dolichols, as cofactors in glycosylation of proteins engaged in plant sexual reproduction, may turn out to be important targets in breeding new crop varieties. Many glycosylated or GPI-modified proteins serve in pathogen response, and dolichols seem to be responsible for preventing ion leakage from membranes. Manipulations of these traits can help improve plant resilience against biotic and abiotic stresses such as massive invasions of pests, drought, and increasing soil salinity in a changing climate. Summarizing, the biology of long-chain isoprenoids is still an open field for both basic and applied research.
